# AT(N), neuronal and glial pathologies underlying neuropsychiatric symptoms and cognition in young‐onset dementia

**DOI:** 10.1002/alz.091889

**Published:** 2025-01-09

**Authors:** Wei‐Hsuan Chiu, Anita M Y Goh, Charles B Malpas, Dhamidhu Eratne, Wendy Kelso, Mark Walterfang, Dennis Velakoulis, Samantha M Loi

**Affiliations:** ^1^ The University of Melbourne, Parkville, VIC Australia; ^2^ The University of Melbourne, Melbourne, VIC Australia; ^3^ Melbourne Neuropsychiatry Centre, University of Melbourne and Melbourne Health, Parkville, VIC, VIC Australia; ^4^ Edith Cowan University, Perth, Western Australia Australia; ^5^ Neuropsychiatry Royal Melbourne Hospital, Parkville, VIC Australia; ^6^ National Ageing Research Institute, Melbourne, VIC Australia; ^7^ The University of Melbourne, Melbourne Australia; ^8^ National Dementia Diagnostics Laboratory, The Florey Institute of Neuroscience and Mental Health, Parkville, VIC Australia; ^9^ Neuropsychiatry, The Royal Melbourne Hospital, Parkville, VIC Australia; ^10^ University of Melbourne, Parkville, VIC Australia; ^11^ Melbourne Neuropsychiatry Centre, University of Melbourne and Melbourne Health, Parkville, VIC Australia; ^12^ Royal Melbourne Hospital & Melbourne Neuropsychiatry Centre, Melbourne Australia

## Abstract

**Background:**

Young‐onset dementia (YOD) refers to the occurrence of dementia symptoms in people under the age of 65. Neuropsychiatric symptoms (NPS) are increasingly considered as the preclinical manifestations of YOD, posing a challenge to differentiate from psychiatric conditions with overlapping symptoms. The aim of this study is to investigate the AT(N), neuronal and glial pathologies underlying NPS and cognition in YOD.

**Method:**

This study used clinical data from the Neuropsychiatry Centre, the Royal Melbourne Hospital, Melbourne, Australia. Sixty‐nine people with YOD were identified and assessed for biomarkers, NPS and cognition (mean age [SD] = 57.6 [7.3], female N = 23 [33%]). Their cerebrospinal fluid and plasma levels of amyloid‐beta, total‐tau (t‐tau), phosphorylated‐tau (p‐tau), neurofilament light chain protein (NfL) and glial fibrillary acidic protein (GFAP) were measured. Common NPS such as depressive and behavioural symptoms were also measured. Cognitive functions were measured by a comprehensive battery of neuropsychological assessments. General linear regressions were used to investigate the associations between biomarkers, NPS and cognition.

**Result:**

Memory recall was correlated with levels of amyloid‐beta (r = .52, 95% conference interval [CI] = [.32, .75]), t‐tau (r = ‐.4, 95% CI = [‐.65, ‐.16]) and p‐tau (r = ‐.34, 95% CI = [‐.58, ‐.11]). Executive function was correlated with levels of NfL (r = ‐.4, 95% CI = [‐.78, ‐.03]). Levels of NfL were further correlated with the severity of stress (r = ‐.41, 95% CI = [‐.73, ‐.11]). No evidence of associations between levels of GFAP and NPS or cognition was observed. Visuoconstruction was correlated with levels of amyloid‐beta (r = .45, 95% CI = [.24, .67]), in which this relationship was moderated by the severity of self‐care problems (F [6, 28] = 5.48, p < .05).

**Conclusion:**

The results suggest that the changes in memory recall are associated with an intricate interplay between AT(N) pathologies in people with YOD. Executive function in YOD may be related to neuronal alterations rather than glial alternations in the brain. Combining biomarker results, the real challenge of self‐care problems in people with YOD observed by their caregivers may serve as a surrogate estimate to predict outcomes in visuoconstruction.

